# Hydroxyapatite Nanoparticles for Improved Cancer Theranostics

**DOI:** 10.3390/jfb13030100

**Published:** 2022-07-20

**Authors:** Saeid Kargozar, Sahar Mollazadeh, Farzad Kermani, Thomas J. Webster, Simin Nazarnezhad, Sepideh Hamzehlou, Francesco Baino

**Affiliations:** 1 Tissue Engineering Research Group (TERG), Department of Anatomy and Cell Biology, School of Medicine, Mashhad University of Medical Sciences, Mashhad 9177948564, Iran; smn.nazarnezhad@yahoo.com; 2 Department of Materials Engineering, Faculty of Engineering, Ferdowsi University of Mashhad (FUM), Azadi Square, Mashhad 9177948564, Iran; farzadkermani73@gmail.com; 3 Programa de Pós-Graduação em Ciência e Engenharia dos Materiais, Universidade Federal do Piauí, Teresina 64049-550, Brazil; websterthomas02@gmail.com; 4 School of Engineering, Saveetha University, Chennai 602117, India; 5 School of Health Sciences and Biomedical Engineering, Hebei University of Technology, Tianjin 065000, China; 6 Genetics Laboratory, Ghaem Hospital, Mashhad University of Medical Sciences, Mashhad 9176699199, Iran; sepidy88@hotmail.com; 7 Institute of Materials Physics and Engineering, Department of Applied Science and Technology (DISAT), Politecnico di Torino, 10129 Torino, Italy

**Keywords:** bioceramics, hydroxyapatite, nanomaterials, cancer treatment

## Abstract

Beyond their well-known applications in bone tissue engineering, hydroxyapatite nanoparticles (HAp NPs) have also been showing great promise for improved cancer therapy. The chemical structure of HAp NPs offers excellent possibilities for loading and delivering a broad range of anticancer drugs in a sustained, prolonged, and targeted manner and thus eliciting lower complications than conventional chemotherapeutic strategies. The incorporation of specific therapeutic elements into the basic composition of HAp NPs is another approach, alone or synergistically with drug release, to provide advanced anticancer effects such as the capability to inhibit the growth and metastasis of cancer cells through activating specific cell signaling pathways. HAp NPs can be easily converted to smart anticancer agents by applying different surface modification treatments to facilitate the targeting and killing of cancer cells without significant adverse effects on normal healthy cells. The applications in cancer diagnosis for magnetic and nuclear in vivo imaging are also promising as the detection of solid tumor cells is now achievable by utilizing superparamagnetic HAp NPs. The ongoing research emphasizes the use of HAp NPs in fabricating three-dimensional scaffolds for the treatment of cancerous tissues or organs, promoting the regeneration of healthy tissue after cancer detection and removal. This review provides a summary of HAp NP applications in cancer theranostics, highlighting the current limitations and the challenges ahead for this field to open new avenues for research.

## 1. Introduction

Cancer is still the second leading cause of death on Earth, with a large number of attempted treatments [1,2,3]. The conventional therapies (e.g., chemotherapy and radiotherapy) usually suffer from critical limitations, most notably systemic toxicity and painful procedures with long recovery times. Even with such conventional treatment, cancer reoccurrence is far too common; for example, the high frequency of bone recurrence (33.3% in the T1 group) has been reported as an initial recurrence site after radical surgery in T1N3 gastric cancer [4]. Therefore, significant efforts have been made to develop and standardize new therapies, including gene-, hormone-, and immuno-therapy. In addition to these approaches, photodynamic therapy and hyperthermia are among the most promising strategies applied for cancer treatment [5]. By utilizing these advanced approaches, clinicians have achieved great successes in the battle against cancer, such as a significant increase in the survival rate of patients [6]. However, truly optimal clinical outcomes still seem out of reach due to a number of factors, including early cancer detection, the need to reduce the toxicity from conventional cancer treatments, and the growing concern over cancer cell therapeutic resistance (e.g., cancer cell chemotherapeutic resistance) [7].

In the era of modern medicine, anticancer materials have significantly aided clinicians in maximizing cancer therapeutic outcomes [8,9,10,11]. Different types of biocompatible materials have been developed to inhibit or kill cancer cells; for example, gold compounds were well documented as metal anticancer drugs [12]. They could be utilized as anticancer gene and drug delivery systems and immunomodulatory substances, as well as photodynamic and hyperthermia agents [13,14,15,16]. Among the biomaterials possessing anticancer activity, hydroxyapatite (HAp) nanoparticles (HAp NPs) offer great opportunities for cancer therapy [17,18]. HAp, with the chemical formula of Ca_10_(PO_4_)_6_(OH)_2_, is considered to be the most stable calcium phosphate (CaP) biomaterial in the biological environment [19,20,21,22]. It should be noted that the biological response of HAp mainly relies on physicochemical properties, such as particle size, crystallinity degree, surface area, morphology, and surface charge [23,24,25]. For instance, a lower degree of crystallinity will result in a high dissolution rate. The theranostic tool loses or leaches a boost of therapeutic agents, which might not always be favorable. Moreover, HAp with various properties with different solubility in the biological environment could improve bone formation or prevent the growth of cells and tissues with cases of dysregulating ion homeostasis [26].

Due to its excellent physico-chemical, mechanical, and biological properties, HAp was first proposed for hard tissue engineering applications in medicine, as has been reviewed elsewhere [27,28]; however, the researchers found that nanometer-sized HAp particles may be applied in other areas of medicine, such as cancer theranostics [29]. The spread of cancer cells to skeletal tissues (mainly consisting of HAp) suggests that there is an important correlation between HAp and cancer cells [5]. Some studies have demonstrated that the administration of HAp NPs inhibits the growth and metastasis of tumor cells, resulting in an improved survival rate of tumor-bearing animals [30]. 

Previously reported studies have also indicated that the chemical structure of HAp provides exceptional possibilities for ion exchange in its anionic (PO_4_, OH) and cationic (Ca) sites (see Table 1) [31]. On this matter, the easy incorporation of magnetic ions (Fe^2+^, Gd^3+^, etc.) into the HAp structure has opened up numerous interesting applications for researchers and scientists who focus on cancer theranostics. As just one of many illustrations, nuclear reactors have been used for neutron irradiation of Sr-doped HAp nanorods, thus generating the radioisotopes ^32^P and ^89^Sr in the HAp structure, which can emit beta radiation for potential applications in bone tumor therapy [32]. Still, these innovative strategies need to be further studied in order to release appropriate and reliable guidelines for preclinical and clinical trials. On the other hand, HAp NPs could serve as a suitable platform for the loading and sustained release of a broad range of anticancer drugs and molecules for targeted cancer therapeutic approaches [33,34,35,36]. It is well understood that targeted therapy enhances the therapeutic effects of anticancer agents both in vitro and in vivo by decreasing systemic toxicity and increasing the local concentration of an anticancer agent. Targeting HAp towards tumor cells has been accomplished via the conjugation and grafting of specific micro- and macro-molecules onto the particle surface, leading to improved HAp uptake by tumor cells [37,38]. For example, HAp NPs were successfully surface-modified by hyaluronic acid to target the delivery of doxorubicin (DOX) toward the mitochondria and nuclei of liver cancer cells [35]. The available literature shows that most experiments combine HAp with other materials (e.g., polymers) to make more versatile and potent anti-tumor composites [39,40,41]. In addition, multivalent ions (e.g., Fe^2+^/Fe^3+^) can be released and regenerated continuously during the interaction of the doped HAp-NPs with the biological fluids. Potential changes during the ion transformation between different valences may be a critical factor in killing cancer cells [42]. Moreover, the release of Fe^2+^/Fe^3+^ ions from Fe-doped HAp compositions plays a critical role in killing cancer cells through the Fenton reaction (Fe^2+^ + H_2_O_2_ → Fe^3+^ + OH^−^ + *OH), which can generate ROS in the tumor environments [42].

Over time, HAp nano-sized particles have been proposed as valuable substances in multimodal molecular imaging. In this manner, superparamagnetic HAp NPs were previously approved as suitable in vivo agents for magnetic resonance (MR) and nuclear imaging [43]. In addition, HAp NPs could be functionalized, either in their pristine or ion-doped forms, by different biocompatible materials (e.g., polymers) and could be utilized for improved bioimaging strategies, including cellular imaging, as well as non-invasive and quantitative visualization of molecular processes [44]. 

In the present review, we provide an in-depth view of the potential of HAp NPs in cancer theranostics (i.e., the combination of therapy with diagnostics) by discussing the previously reported experimental data available from the literature. In vitro and in vivo studies are included in this review to present a complete picture and unify the thoughts concerning the real potential of HAp NPs in treating and imaging solid tumors. Finally, the key opportunities and challenges that lie ahead for the pre-clinical and clinical use of HAp NPs in cancer precision medicine are discussed in an attempt to open new avenues for research. 

**Table 1:** Ionic exchange/doping in the hydroxyapatite (HAp) structure, useful for cancer therapy and diagnostics.

Element	Ionic Charge(s)	Advantages	Ref(s)
Fluoride (F)	1−	-Provides the possibility of medical imaging of soft tissues (e.g., breast cancers) using positron emission tomography (PET)/computer tomography (CT)	[45]
Silicon (Si)	4− 4+	-Promotes drug loading efficiency, good biocompatibility, and biodegradability	[46]
Sulfur (S)	2−, 2+, 4+, 6+	-In the form of sulfate, may make a pH-responsive platform for efficient tumor-targeting drug delivery	[44]
Selenium (Se)	2−, 4+, 6+	-Exerts a strong cytotoxic activity against prostate (PC3) and breast (MDA-MB-231) cancer cells-Inhibits human osteosarcoma cell growth via inducing apoptosis	[47,48]
Strontium (Sr)	2+	-The radioactive isotope strontium-89 renders luminescent properties	[32]
Barium (Ba)	2+	-Acts as a suitable computed tomography (CT) contrast agent	[49]
Hafnium (Hf)	4+	-Induces the formation of ROS and cell apoptosis (in vitro and in vivo) after being exposed to ionizing radiation (e.g., gamma rays)	[50]
Zinc (Zn)	2+	-Enhances the radiation of breast cancer cells	[51]
Copper (Cu)	1+, 2+	-In the form of a nanocluster, it can impart luminescent properties to HAp, providing a suitable material for cancer imaging	[52]
Iron (Fe)	2+, 3+	-Imparts superparamagnetic behavior (useful in MR cancer imaging) and causes damage to mitochondria and cancer cell membranes-Fenton reaction-based cancer treatment.	[42,53]
Zirconium (Zr)	4+	-Accumulates and internalizes in the lung cancer cell line A549	[54]
Europium (Eu)	3+	-Acts as a fluorescent probe and is thereby suitable for tracking cancer cells	[55]
Terbium (Tb)	3+, 4+	-Provides photoluminescence properties	[55]
Samarium (Sm)	3+	-Suitable for bioimaging, drug delivery, and as an MRI contrast agent	[56,57,58,59,60]
Neodymium (Nd)	3+, 4+	-Exhibits near-infrared emission at 670 nm after excitation at 410 nm, unraveling its theranostic capabilities -Excellent compatibility with mammalian cells	[59]
Gadolinium (Gd)	3+	-Great potential for applications as an MR T1 contrast agent and drug carrier for cancer therapy	[61,62]
Vanadium (V)	2+, 3+, 4+, 5+	-Causes strong cytotoxicity against human bone cancer cells	[63]

## 2. Hallmarks of Cancer: A Call for Theranostic Nanoparticles

Six major hallmarks of cancer were first introduced by Hanahan and Weinberg in 2000 [64], which can be listed as follows: (I) sustaining cellular proliferative signaling; (II) insensitivity to anti-growth cellular signals; (III) resistance to cellular apoptosis; (IV) cellular replicative immortality; (V) stimulated angiogenesis; and (VI) tissue cancer cell invasion and metastasis [65]. In addition, the reprogramming of cellular energy metabolism and evading immune system destruction were introduced as two additional emerging hallmarks to those listed above [66]. Indeed, all of these phenomena have been identified as key players in the remarkable diversity of neoplastic diseases as they affect genomic stability. 

NPs such as HAp nanostructures were previously proposed to simultaneously target some of the hallmarks of cancer and for the safe and effective transport and release of multiple cytotoxic agents [67]. For example, HAp NPs can inhibit cancer cell growth, migration, and invasion via downregulating the FAK/PI3K/Akt cell signaling pathway, both in vitro and in vivo [68]. HAp NPs can also cause oxidative stress-induced apoptosis in cancer cells by lysosomal and mitochondrial-dependent pathways, including caspase 3 [69]. Prior experimental studies have confirmed the suitability of drug-loaded multi-modal HAp NPs as effective substances for tumor metastasis resisting therapy (TMRT) and tumor metastasis targeting therapy (TMTT) [70]. Still, there is a relative paucity of studies in the literature focusing on targeting and imaging the multiple hallmarks of cancer by applying nano-sized HAp particles, either in the pristine or in the functionalized and drug-loaded types. 

## 3. HAp Structure Fits Therapeutic Applications 

**Figure 1:**
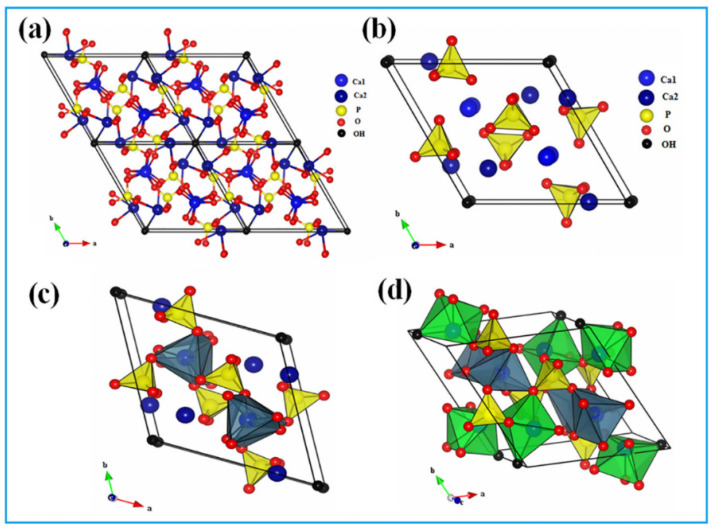
Schematic representation of (**a**) the HAp unit cell (001) plan; (**b**) the distribution of octahedral site of PO_4_^3−^ in the HAp structure; (**c**) the distribution of the tetrahedral sites of PO_4_^3−^ and sequence of the octahedral sites; and (**d**) the tetrahedral and octahedral sites of PO_4_^3−^ in the HAp structure. Reproduced with permission from [77].

HAp can crystallize in the hexagonal and monoclinic systems with a space group of P6_3_/m and P2_1_/b, respectively [71]. The lattice parameters of HAp in the hexagonal systems are a = b = 9.432 Å, c = 6.881 Å, and γ = 120° [72], while the lattice parameters of the monoclinic structure of HAp are a = b/2 = 9.421 Å, c = 6.881 Å, and γ = 120° [73]. The hydroxyl (OH) groups are orientated differently in the two structures of HAp; the adjacent OH groups point in the opposite direction in the hexagonal structure, while the OH groups in a given column point in the same direction in the monoclinic structure [74]. As the hexagonal structure of HAp is found in natural bones, more attention has been paid to its relevant physico-chemical, mechanical, and biological properties [75]. For example, Posner et al. [76] first refined the crystal system of HAp with the hexagonal system, suggesting a distribution of 10 Ca^2+^, 6 PO_4_^3−^, and 2 OH^−^ in each unit cell of HAp. In the HAp unit cell, calcium ions (Ca^2+^) are observed in two different sites, i.e., the accurately aligned column (Ca1) or with the adjacent OH groups oriented in the opposite direction (Ca2). PO_4_^3−^ tetrahedral and octahedral arrays remain in the inside and the periphery of the unit cells, where P^5+^ is in the center and O_2_ is on the top of the tetrahedrons. Ca^2+^ ions are interspersed with the PO_4_^3−^ tetrahedrons, and the OH groups are located at the edge of the unit cell (Figure 1). 

Creating structural defects in HAp is a common approach during its synthesis process; the incorporation of therapeutic ions (e.g., Sr^2+^) and heat treatments via high temperature are among the most widespread strategies [78]. The defects in HAp can be categorized into distinct types, including oxygen vacancies (O removal from O-H or PO_4_ groups), O-H group vacancies, interstitial protons, and impurities (impurities in carbonated groups are common) [78,79]. Prior computational studies have investigated the effect of defects on the properties of HAp and compared them with experimental studies [80,81,82]. Deliberately created defects may change the physico-chemical properties of HAp (the band-gap, surface charge, and area, as well as the ion release/uptake) and thereby improve its biological behavior (e.g., improve osteogenesis) [83]. 

The use of nano-scaled HAp has attracted much attention in cancer theragnostic strategies with regard to its ability to easily become internalized into cancer cells and support the targeted delivery of anticancer substances [84]. Up to now, a series of chemical methods have been developed to prepare HAp NPs with a defined microstructure. All of the synthesis routes could be classified into five groups, including: (I) dry methods (solid-state and mechanochemical routes); (II) wet methods (chemical precipitation, hydrolysis, sol-gel, hydrothermal, emulsion, and sonochemical routes); (III) high-temperature processes (combustion and pyrolysis methods); (IV) approaches based on biogenic sources (biogenic wastes); and (V) combination procedures [85]. By applying the aforementioned methods, HAp nanostructures with different sizes and shapes can be produced, including spheres, rods, needles, flakes, flowers, mesoporous spheres, bowknots, dumbbells, etc. [85]. It should be mentioned that the size, crystallinity, and morphology of Hap NPs are among the most critical factors affecting their anticancer properties [86,87]. The mentioned properties effectively affected the anionic and cationic sites of HAp containing calcium and phosphorous ions, and also hydroxyl, amino, and carboxyl groups. The positive and negative surface charge of HAp, which depends on its physicochemical properties, significantly affects the interaction of HAp with a biomolecule.

**Figure 2:**
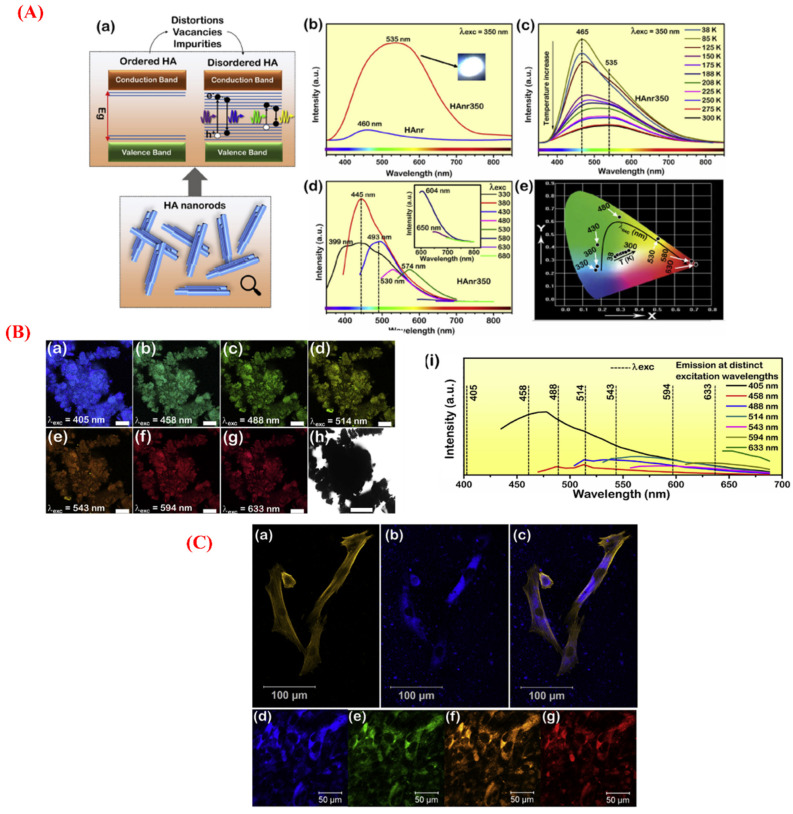
(**A**) The optical behavior of HAp nanorods (HApnr): (a) the general model for self-activated fluorescence in the nano-sized particles, in which the energy levels in the forbidden zone are minimized while the presence of defects and the elimination of impurities lead to a higher disorder in the HAp structure, allowing radiative recombination of e’–h^•^ pairs at defect energy levels between the valence band (VB) and conduction band (CB); (b) the emission spectra of HApnr and HApnr350 samples by laser excitation at λ_exc_ = 350 nm; (c) the temperature-dependent photoluminescence of HApnr350 sample by laser excitation at λ_exc_ = 350 nm; (d) the excitation dependence of the HApnr350 sample probed by the spectrofluorometer at room temperature; and (e) the Commission Internationale de l’Éclairage (CIE) chromaticity diagram. (**B**) The optical response of the HAnr350 species obtained by confocal microscopy, monitored by exciting at λ_exc_ = 405, 458, 488, 514, 543, 594, and 633 nm (a, b, c, d, e, f, and g, respectively): (h) the bright-field micrograph of the power analyzed; scale bar = 200 mm and (i) the corresponding emission spectra obtained by exciting at distinct wavelengths. (**C**) The confocal imaging of human dermal fibroblast (HDFn) cells incubated with the HAnr350 sample (320 mg/mL) for 48 h: (a) the cytoskeleton labeled with Alexa Fluor 532 Phalloidin (λ_exc_ = 514 nm); (b) the fluorescence image exhibiting blue fluorescent HAp NPs (λ_exc_ = 405 nm); and (c) merged images. (d–g) The multiple excitation/emission imaging of intracellular the HAnr350 NPs (λ_exc_ = 405, 488, 543, and 594 nm, respectively). Reproduced with permission from ref [89].

In addition to therapeutic strategies, structural modifications to HAp NPs provide great opportunities for producing self-activated fluorescent substances, which are excellent intracellular imaging tools for both prokaryotic and eukaryotic cells [88]. In a recently published experiment, Machado et al. designed biocompatible and multicolor fluorescent HAp nanorods for cell-imaging applications [89]. For this aim, the HAp nanorods were synthesized using chemical precipitation followed by heat treatment at a relatively low temperature (350 °C), exhibiting intense and tunable fluorescence emissions. The internalization of the HAp NPs into human dermal fibroblast neonatal (HDFn) cells was successfully recorded because localized particles at regions near the nucleus structure were observed. The suitability of the HAp NPs for multicolor cell imaging was demonstrated by observing the intense fluorescence emissions of blue, green, yellow, and red colors in HDFn cells incubated with the HAp NPs and imaged at wavelengths of 405, 488, 543, and 594 nm, respectively (Figure 2). The emitted colors changed from blue to red and also white, depending on the applied temperature, the source of optical measurements, and the excitation wavelengths. The authors stated that this behavior was related to structural order–disorder effects induced by defects, including impurities (CO_3_^2−^, H_2_O, and NH_4_^+^), distortions in the structural clusters, and vacancies (Ca, OH). Furthermore, the elimination/decomposition of the impurities trapped in the HAp lattice could be considered as a critical step for improving the radiative recombination of e’–h^●^ pairs between defect-related energy levels. 

In addition to structurally modified HAp nanostructures, HAp NPs can also be utilized for cancer cell imaging through different well-established approaches, including: (I) the combination with organic fluorophores; (II) doping with various lanthanide ions; and (III) the manufacturing of composites with other inorganic nanomaterials (e.g., carbon-quantum and chalcogenide-quantum dots) [90,91]. 

## 4. Interactions of HAp NPs with Cancer Cells

**Figure 3:**
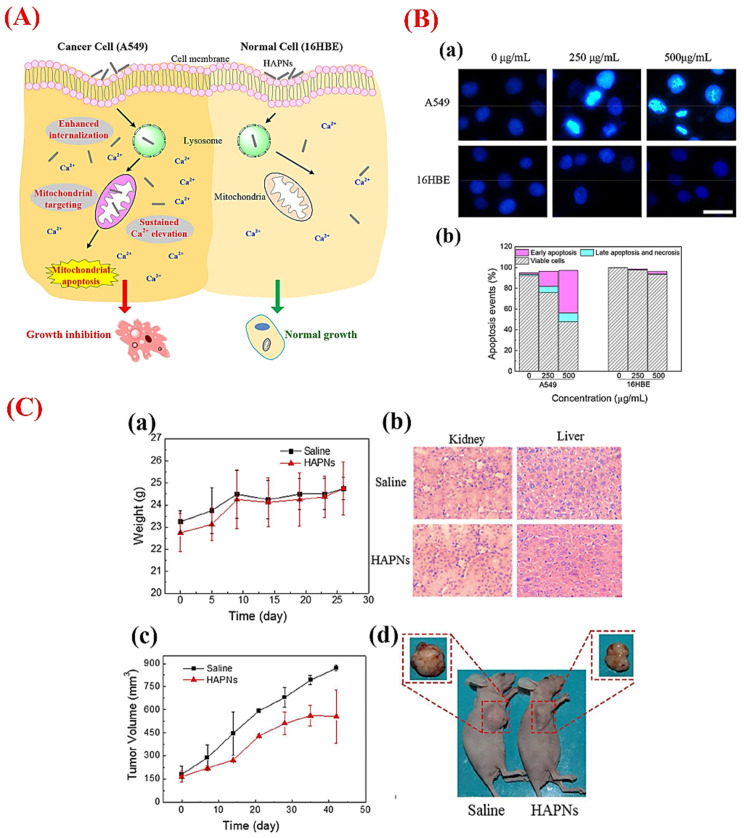
(**A**) HAp NPs can induce cytotoxicity in cancer cells (adenocarcinoma human alveolar basal epithelial cells, A549 cells) through mitochondria-mediated apoptosis due to higher internalization as compared to normal cells (human bronchial epidermal cells, 16HBE cells). (**B**) Fluorescent micrographs of cells (a) before and after treatment with different doses of HAp NPs for 48 h (scale bar: 20 µm) and (b) Annexin V-FITC/PI double staining analysis of the cells showing apoptosis ratios in A549 and 16HBE cells post-treatment with HAp NPs. (**C**) In vivo tumor inhibition efficacy of HAp NPs: the effect of the HAp NPs treatments on changes in the body weight of healthy nude mice at a dosage of 40 mg/kg (three times a week) (a); histology evaluation (hematoxylin and eosin staining) of kidney and liver samples from healthy nude mice treated with saline or HAp NPs (40 mg/kg) on day 26 (magnification 200×) (b); tumor growth curves of nude mice bearing xenografted A549 cells treated with 40 mg/kg of HAp NPs three times a week (c); and macroscopic images of mice and excised tumors from saline or HAp NPs-treated groups at the time of sacrifice (d). Reproduced with permission from ref. [98].

Several in vitro and in vivo studies have demonstrated the inhibitory effects of HAp NPs on the growth and migration of cancer cells [92,93,94,95]; a selection of studies is reported in Table 2. Some physico-chemical properties of HAp-NPs, including the shape, size, and crystallinity, are recognized as key determinants in their anticancer activities [87,96,97]. The properties of HAp-NPs have a particular effect on the adsorption capacity of the drug/gene/protein and solubility in physiological fluids; accordingly, the interaction of HAp with cancer cells is related to the HAp nanostructured properties. For example, Dey et al. showed that rod-shaped HAp NPs with a 22 nm crystallite size and 0.43% crystallinity degree significantly prohibited the proliferation of colon cancer HCT116 cells [86]. HAp NPs exerted their anticancer effects by the up/down-regulation of specific molecules and signaling pathways in cancer cells. Sun et al. previously reported that rod-shaped HAp NPs (10 and 50 nm in width and length, respectively) enter human lung cancer cells (A549) by caveolae-mediated endocytosis through lysosome trafficking [98]. In A549 cells, the translocation of the NPs to mitochondria was recorded. Additionally, HAp NPs (at concentrations of 250 and 500 µg/mL) caused a sustained increasing of the intracellular calcium concentration in cancerous cells, while transitorily increasing the Ca^2+^ levels in normal bronchial epithelial cells (16HBE). HAp nanorods induced apoptosis in A549 cells, and higher dosages (500 µg/mL) caused more apoptotic cells with no significant adverse effects on normal cells. The efficacy of HAp NPs was also demonstrated in a lung cancer xenograft model as they yielded a moderate reduction in tumor size without any overt toxicity in the treated animals (Figure 3).

It was also stated that HAp NPs induce apoptosis (programmed cell death) in tumor cells by promoting p53 expression and its downstream genes [99]. Tang et al. observed that HAp, via the activation of caspase-3 and -9 (but not caspase-8), inhibits cell proliferation and induces apoptosis in different cancer cells (gastric cancer cells [MGC80-3], cervical adenocarcinoma epithelial cells [HeLa], and hepatoma cells [HepG2]) [100]. In another report, the anticancer effects of HAp NPs were examined on human glioma U251 and SHG44 cells both in vitro and in vivo [101]. HAp NPs at dosages of 120 mg/L and 240 mg/L induced apoptosis 48 h post-incubation. Moreover, the cellular tumor growth capacity was prohibited after the injection of HAp NPs in vivo in a rat model, and the related side effects of the chemotherapeutic drug 1,3-bis(2-chloroethyl)-1-nitrosourea (BCNU) significantly decreased if it was administered concurrently with HAp NPs. A reduction in the expression of c-Met, SATB1, Ki-67, and Bcl-2 protein, SLC22A18, and caspase-3 was attributed to the anticancer impact of HAp NPs. In other experimental studies, HAp NPs were confirmed to produce and cause the intracellular accumulation of reactive oxygen species (ROS) that damage the DNA of cancer cells [102]. High amounts of HAp NPs can be internalized by endocytosis in cancer cells around the endoplasmic reticulum, inhibiting protein synthesis via decreasing the binding of mRNA to the ribosomes in cells by their high ribosome adsorption capacity and arresting cell cycle in the G0/G1 phase [92]. It is worth mentioning that the anticancer effect of nano-sized HAp may be further increased by the incorporation of specific elements (e.g., Se) into its structure [48,103].

Although the existence of a relationship between the physico-chemical properties and the anticancer effects of HAp-NPs has been demonstrated, the generalization of such a relationship is still far from being achieved. This is due to the multiplicity of factors involved, the mutual synergy between them, and the variability associated with the response of different cancer cell types.

**Table 2:** HAp nanostructures applied for cancer theranostic strategies.

HAp Nanostructure	Cancer Type	In Vitro/In Vivo	Imaging/Therapy	Remarks	Ref
Strontium (Sr)-doped HAp nanorods	Bone	In vitro	Therapy	Generation of the radioactive isotopes of strontium-89 and phosphorus-32 through neutron irradiation leads to the treatment of bone tumors while regenerating the affected region	[32]
Hafnium (Hf)-doped HAp nanoparticles	Lung	In vitro/In vivo	Therapy	Generation of reactive oxygen species (ROS) after gamma irradiation and inducing apoptosis	[50]
Catechins-modified selenium-doped HAp nanoparticles	Bone	In vitro	Therapy	Stimulating the apoptosis of human osteosarcoma MNNG/HOS cell lines via generation of ROS, inducing the overexpression of caspase-3, p53, and Bax while downregulating cox-2 and PTK-2, with no side effects on human bone marrow stem cells	[104]
Se-doped HAp/chitosan bio-papers	Bone	In vitro/In vivo	Therapy	Overgeneration and accumulation of ROS, inducing apoptosis of HCS 2/8 chondrosarcoma and SJSA osteosarcoma cells through overexpression of Bax, caspase-3, JNK activation, and STAT3 inhibition and lessening the size of the tumor in a patient-derived xenograft (PDX) mouse model	[103]
Selenium (Se)-substituted HAp nanoparticles	Hepatocellular carcinoma	In vitro/In vivo	Therapy	Promoted the survival rate of nude mice, inducing tumor necrosis, reduced toxicity on liver and kidney functions	[105]
Europium (Eu)-doped nanoHAp	HeLa cells	In vitro	Imaging/therapy	Strong fluorescence properties, biocompatible, good delivery, and sustained drug (5-fluorouracil) release	[106]
Eu-doped calcium-deficient HAp core functionalized with cyclodextrin and cucurbituril	HeLa cells	In vitro	Imaging/therapy	Luminescent properties, controlled and prolonged drug (5-fluorouracil) release, the efficient killing of HeLa cells (over 80%)	[107]
Ag/Fe co-doped HA	HeLa cells	In vitro	Therapy	Targeted drug (5-fluorouracil) delivery to the tumor site, controlled drug release, and the killing of the cancerous cells with no cytotoxic effect on the normal cells (HEK-293) and without any infections	[108]
Neodymium (Nd)- doped HAp nanoparticles	Colon	In vitro/In vivo	Imaging/therapy	Early stage diagnosis of tumors, targeted colon tumor therapy, and monitoring	[60]
Eu-HAp-loaded PLGA NPs with foliate decoration	Breast	In vitro	Imaging	Promoted fluorescent properties, targeted imaging of MCF-7 cells at early stages, biocompatible and photostable	[109]
Eu/Gd co-doping HAp nanocrystals	Liver, spleen, heart, lung, kidney	In vitro/In vivo	Imaging	Biocompatible, biodegradable, improved luminescence and fluorescence properties for cell imaging	[90]
Eu (or Tb)-doped HAp nanorods	A549 cells (lung cancer) and HeLa cells	In vitro	Imaging	Biocompatible, excellent red or green luminescent properties, converting to hydrophilic particles with surfactant Pluronic F127 and applications for live cell imaging	[110]

## 5. HAp Nanostructures for the Delivery of Anticancer Drugs

Nanostructured HAp has several merits for consideration as a drug carrier in cancer theranostic applications [111]. These advantages could be summarized as follows: (I) HAp could be prepared with relatively similar properties (e.g., chemistry, crystalline structure, and size) to the major constituents of the targeted tissues (especially bone); (II) HAp alone is relatively stable under variations of the solution/environment (e.g., pH, temperature)—unless produced in the form of NPs—and does not swell or change porosity, preventing the burst release of drugs; (III) HAp can be surface-functionalized with both positively and negatively charged molecules; and (IV) HAp might be tailored as NPs with promising electrical, mechanical, magnetic, and optical properties via doping with different elements [72]. 

Therefore, HAp NPs have been extensively evaluated for the loading and delivery of a series of natural and synthetic anticancer agents, aimed at managing both hard- and soft-tissue related cancers [112,113]. Mesoporous HAp NPs represent an attractive class of drug delivery platforms for loading various anticancer cargos, including chemicals, small molecules, and generic drugs. These systems show promise in cancer therapeutic approaches due to their excellent ability for drug adsorption, storage, and release [114]. In fact, HAp NPs with mesoporous structures (i.e., pore sizes in the range of 2– 50 nm) possess high pore volumes and adequate pore sizes for storing therapeutic molecules and targets as compared with their macroporous (pore sizes > 50 nm) and microporous (pore sizes <2 nm) counterparts [115,116]. Moreover, it is feasible to prepare smart drug delivery systems based on HAp/polymer nanostructures as they could be tailored to be responsive to external stimuli, including temperature, pH, light, ultrasound, enzymes, etc. [117,118,119,120,121]. 

Regarding the literature, pH-sensitive nanostructures constitute a large part of the experimental studies; for example, Li et al. developed a core-shell nano-carrier based on mesoporous HAp NPs (as the core) and polyacrylic acid (PAA) (as the shell) for effectively loading and delivering the anti-cancer drug doxorubicin (DOX) [122]. Nanostructured hollow HAp was also mentioned as a suitable material for enhanced anticancer drug delivery toward cancer cells [123]. However, although utilizing nano-sized hollow HAp structures for the delivery of anticancer substances seems promising in the beginning steps, much more attention is currently being paid to larger-scale hollow micro-sized HAp structures (e.g., microspheres) [29,124]. More recently, Liu et al. reported the production of flower-like HAp spheres (FHAPS), consisting of hierarchical nanosheets synthesized by the one-pot calcium carbonate (CaCO_3_) transformation method for the effective delivery of doxorubicin (DOX) and siRNA into breast cancer cells [125]. The obtained results confirmed the appropriate loading of DOX (9.1%) and siRNA (2.0%), as well as the effective delivery of anticancer agents into drug-resistant breast cancer cells (MCF-7/ADR cells). Moreover, the degradation of the drug-loaded samples occurred after 48 h in cancer cells. It is worth mentioning that coating HAp with biopolymers can improve the bioactivity and controlled release of anticancer drugs [126].

## 6. Doped HAp NPs for Cancer Theranostics

**Figure 4:**
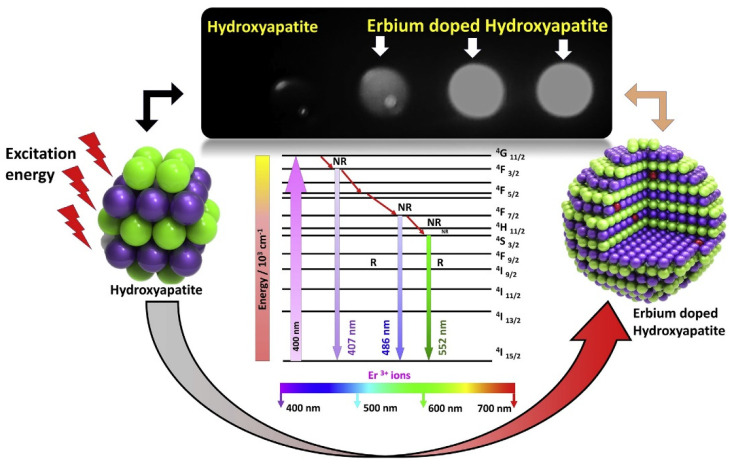
Schematic illustration of erbium (Er)−doped hydroxyapatite (HAp) particles as a bioactive luminescent platform useful for cancer imaging. Reproduced with permission from ref. [128].

Current chemotherapy treatments usually result in cytotoxicity to bone marrow cells and immune cells due to severe side effects as such drugs need to be administrated at high drug doses. The ability to support normal cell growth while inducing cancer cell death is a key feature of the next generation of anticancer substances. Accordingly, HAp NPs doped with a series of therapeutic ions have significant relevance for cancer treatment/diagnosis and have been developed and applied for cancer theranostic applications [31]. As mentioned previously, HAp particles may undergo some changes in their crystal structure, crystallinity, surface charge, and solubility after being doped with therapeutic ions [31]. For instance, the impact of the calcination temperature and Eu^3+^ doping content was previously assessed as per the luminescence properties and phase composition, crystal size, and crystallinity of Eu^3+^-doped HAp NPs [127]. The thermal treatment (600 °C) and 2% Eu^3+^ doping content were optimized for fabricating nanocrystalline Eu-doped HAp (20–40 nm in diameter) with strong luminescence. In another study, luminescent erbium (Er^3+^) ions at different concentrations (0.1, 0.25, 0.5, and 1.0 mol%) were added to HAp nanocrystals (Er-HAp) to develop a nontoxic luminescent agent for biomedical imaging applications [128]. The results showed that 1.0 mol% Er-doped HAp nanocrystals (<50 nm size distribution) had high-efficiency light emission after being excited at 400 nm (Figure 4).

Specific magnetic elements (e.g., Fe and Mn) and oxides (e.g., Fe_3_O_4_, Fe_2_O_3_, and γ-Fe_2_O_3_) were also added to HAp, making NP-based hyperthermia therapies possible [129,130]. Apart from the magnetic elements, researchers can take advantage of other therapeutic ions in the HAp structure; for example, selenium (Se)-substituted HAp NPs showed promising properties for reducing human osteosarcoma cell survival without any adverse effects on human BMSCs [131]. 

The use of radioactive isotopes in cancer therapy shows some limitations due to their toxicity when dissociated from their chelate and deposited into healthy tissues [132]. Therefore, their incorporation into biocompatible carriers (such as HAp nanostructures) has been proposed as an innovative and promising approach in cancer theranostics. Until now, a series of radioactive isotopes was successfully added to HAp NPs, enabling the simultaneous treatment and imaging of tumors. Radioisotopes of strontium-89 (^89^Sr), phosphorus-32 (^32^P), and gadolinium-159 (^159^Gd) are some examples of elements incorporated into the HAp structure and evaluated for their anticancer capacity [32,60]. These doped HAp NPs could act as internal radiation sources through the emission of beta and gamma radiation, leading to a reduction in treatment-resistant tumors.

In addition to the management of bone tumors, the theranostic potential of doped HAp NPs has also been examined for other cancer types, including breast, lung, colon, and liver tumors [49,62,133]. As an illustration, Victor et al. synthesized neodymium-doped HAp NPs by a facile co-precipitation method in order to prepare a smart theranostic nanoplatform for colorectal cancer [60]. The incorporation of neodymium (Nd) into HAp NPs provides near-infrared fluorescence ability. The authors surface-functionalized the samples with alginic acid by using aminopropyltriethoxysilane (APTS) and 1-ethyl-3-(-3-dimethylaminopropyl) carbodiimide hydrochloride (EDC) to impart pH responsiveness to the nanoplatforms that could thus deliver the model drug 4ASA to the colon after oral administration. The results demonstrated that this system simultaneously imaged cancer and targeted drug delivery to colon cancer cells, holding promise for early tumor detection and the treatment of colorectal cancer.

**Figure 5:**
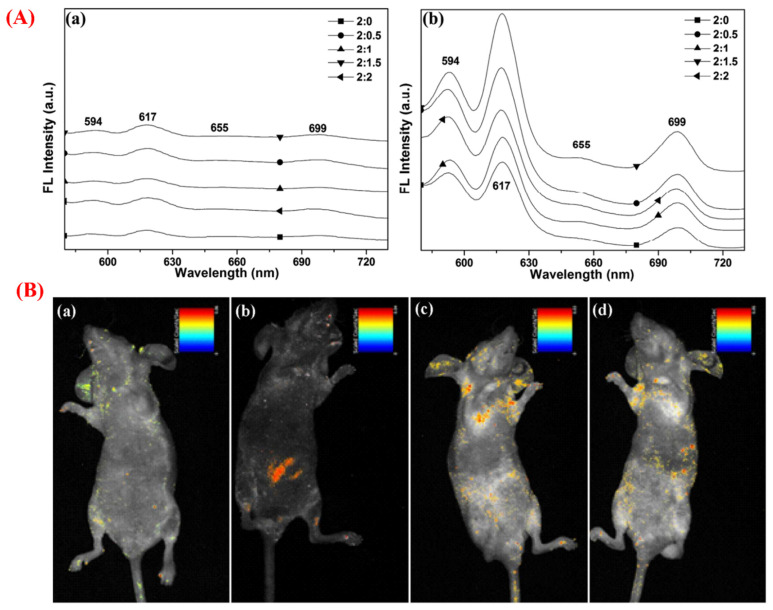
(**A**) Graphs reporting the emission spectra of non-calcined HAp: Eu/Gd excited at 273 and 394 nm ((a) and (b), respectively). (**B**) The in vivo fluorescent imaging of HAp: Eu/Gd (2:1.5) nanocrystals with no calcination in BALB/c-nu mice: (a) with no injection, (b) with injection in the enterocoelia, (c) five minutes post-vein injection, and (d) 1.5 h post-vein injection. Reproduced with permission from ref. [90].

Generally, two main approaches are being applied to improve the luminescence intensity of doped HAp nanostructures. An increase in dopant content in the HAp structure is proposed as the first method; however, this may lead to fluorescence quenching and greater cytotoxicity. The second strategy is related to calcination at high temperatures to increase the luminescence intensity by improving the HAp particle crystallinity as well as enhancing the diffusion of dopants into the HAp crystalline lattice. However, calcination may increase the size and agglomeration of HAp crystals, interfering with cellular uptake. As an alternative approach, co-doping of HAp NPs has been investigated to improve luminescence intensity. For example, HAp nanocrystals were co-doped with Eu^3+^ (2 mol%) and Gd^3+^ (0.5, 1, 1.5, 2 mol%) ions for boosting luminescence properties, making them suitable as imaging agents in vitro and in vivo [90]. The main reason behind this elevated luminescence property (up to about 120%) was attributed to the energy transfer from Gd^3+^ to Eu^3+^ (^6^P_J_ of Gd^3+^→ ^5^H_J_ of Eu^3+^) under 273 nm and the possible combined effect of the cooperative up-conversion and the successive energy transfer under 394 nm (Figure 5A). The synthesized samples showed good biocompatibility and hemocompatibility with a blood circulation time of at least 3 h after vein injection into BALB/c-nu mice. In an intracellular acidic environment, the Eu/Gd co-doped HAp nanocrystals exhibited suitable biodegradability (about 65% after 72 h). Intraperitoneal and tail vein injection of the Eu/Gd co-doped HAp nanocrystals caused a fluorescent signal in BALB/c-nu mice; the HAp nanocrystals accumulated mainly in the liver (about 160 ± 37 nmol/g) due to reticuloendothelial system (RES) uptake (Figure 5B). Regarding the active surface of HAp and the possibility of its easy modification by tumor-targeting ligands, the authors concluded that Eu/Gd co-doped HAp nanocrystals might actually have a tumor-targeting function.

HAp NPs have also received great attention in targeted magnetic hyperthermia for cancer treatment [134,135]. Hyperthermia, relying on the mild elevation of temperature to 40–43 °C upon application of an external magnetic field, is a well-established technique for inducing cancer cell death without affecting healthy cells, as well as enhancing the effects of radiotherapy and chemotherapy [136,137]. Furthermore, cancer-targeting magnetic HAp NPs can accumulate in the tumor site by applying an alternating magnetic field. Additionally, the ability of magnetic HAp NPs was previously proven for synergistic chemo-hyperthermia therapy. At this point, magnetic nanocomposites of cobalt ferrite/HAp were synthesized by the microwave-assisted wet precipitation method and loaded with the chemotherapeutic drug (5-fluorouracil, FU) [138]. The prepared nanocomposites showed ferromagnetic behavior with a magnetic saturation value of about 2.5–8.2 emu/g. They were able to increase the temperature (causing a hyperthermic effect) within a short time (43 °C in 4.5 min) as well as release the encapsulated FU after exposure to an alternating magnetic field. Furthermore, the samples exhibited an appropriate capacity to suppress the growth of osteosarcoma cells (MG63), indicating their effectiveness for synergistic chemo-hyperthermia therapy. It should be noted that the co-doping of HAp NPs with Ag^+^ and Fe^2+^ was also evaluated for treating malignant tumors and overcoming bacterial infections [108]. Moreover, there are great opportunities for HAp NP-based brachytherapy (i.e., a sort of internal radiation therapy) thanks to its excellent biocompatibility and tunable degradability. 

**Figure 6:**
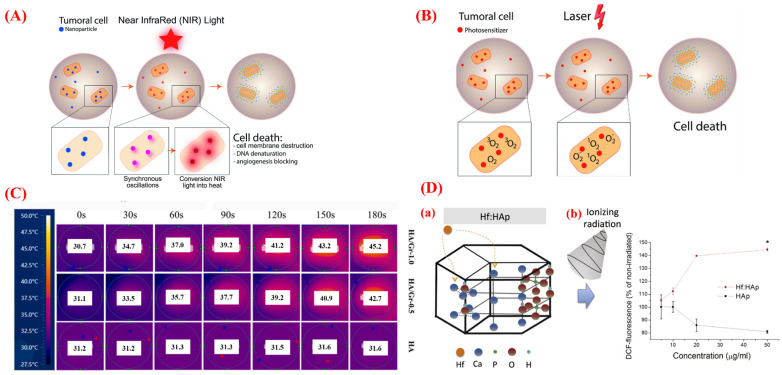
(**A**) Photothermal therapy (PTT) mechanism. (**B**) Photodynamic therapy (PDT) mechanism. (**C**) The effect of NIR on increasing the temperature of HAp/gelatin/graphene scaffold and the role of increasing the concentration of graphene on increasing the temperature in PTT. (**D**) (a) The structure of the doped HAp-NPs with Hf for potential use in PDT; (b) the ROS generation of HAp-Hf in the non-irradiated and gamma rays (5 Gy) conditions. The DCF-fluorescence intensity significantly increased in the irradiated samples doped with Hf. Reproduced with permission from refs [50,142,144].

Photothermal therapy (PTT) and photodynamic therapy (PDT) are among the newest therapies for tumor treatments under external irradiation sources [139,140]. In PTT or strategies involving NP–mediated hyperthermia, the NPs are illumined with a suitable near-infrared (NIR) wavelength (the electromagnetic radiations are NIR with a wavelength within 780–2526 nm) [141]. The conduction band electrons of the treated NPs undergo synchronized oscillations under the effect of NIR. It causes the transformation of light into heat (45–50 °C) and kills cancer cells through cell membrane destruction, tumoral DNA denaturation, and angiogenesis blocking mechanisms (Figure 6A) [142]. In PDT, a photosensitizer (PS) is activated with a special wavelength illumination (620–690 nm) [143]. Reactive oxygen species (ROS) and free radicals are generated during a photochemical reaction between the exposed PS and the oxygen. The known mechanisms behind cancer cell death in PDT are direct (necrosis and apoptosis) and indirect (microvascular damage and antitumor immune responses) during the interaction between exposed PS with a specified light wavelength and oxygen (^3^O_2_), which can create cytotoxic singlet oxygens (^1^O_2_ and O^2−^) (Figure 6B) [142]. It should be noted that the temperature that can be reached during these therapies should be controlled to avoid tissue damage. In this scenario, Zhang et al. investigated the photothermal effect of 3D-printed HAp NP scaffolds containing gelatin and graphene nanoplatelets [144]. They stated that a biocompatible system containing HAp and graphene could convert NIR into heat with excellent efficiency in accelerating bone regeneration after photothermal treatment of malignant bone tumors. Their results showed that the growth of MC3T3-E1 cells was significantly increased after three cycles of treatment at mild photothermal treatment (40–43 °C). The results showed that the detected temperature following the exposure of HAp-NPs to NIR was 31.6 and 45.2 °C for the pure HAp and the HAp scaffolds containing 1 wt.% graphene. The used system shows promise for NP-mediated hyperthermia (PTT) (Figure 6C). Furthermore, Chen et al. proposed hafnium-doped hydroxyapatite (Hf: HAp) for enhancing ROS amounts inside tumor cells after ionizing radiation [50]. Their in vivo and in vitro results indicated the effectiveness of Hf: HAp-NPs for killing the A549 lung cell line and inhibiting tumor growth in animals. They observed that the ROS level of HAp and Hf (15 mol%) is similar in the non-irradiated condition of the particles. When the particles were exposed to the excitation of gamma rays (5 Gy), the DCF-fluorescence intensity significantly increased with the increasing of the Hf dosage compared to the non-irradiated groups (Figure 6D). Their finding suggested that the PDT method using HAp-NPs as a compatible substrate doped with Hf has a great potential for cancer palliative treatment.

## 7. Surface-Modified HAp Nanosystems for Targeted Therapy

The desirable loading and delivery of anticancer substances to tumor cells face several limitations, including poor targeting efficiency and lack of drug solubility. To address these restrictions, several innovative approaches have been applied for preparing HAp nanostructures as smart theranostic platforms, which can target cancer cells without damaging normal cells. Among them, the surface functionalization of HAp nanostructures is recognized as one of the most appealing strategies that can support sustained anticancer drug release [145]. For example, HAp NPs functionalized with different carboxylic acids (lactic acid, tartaric acid, and citric acid) were applied to effectively load and deliver curcumin to MCF-7 cells [146]. Carboxylic modifiers could improve the binding affinity between curcumin molecules and the HAp surface based on the electrostatic interactions of opposite charges between the drug and the bioceramic particles. The outcomes clarified that surface-functionalized HAp NPs have higher anti-proliferating and apoptotic activities against the cancer cells as compared to the un-modified samples.

**Figure 7:**
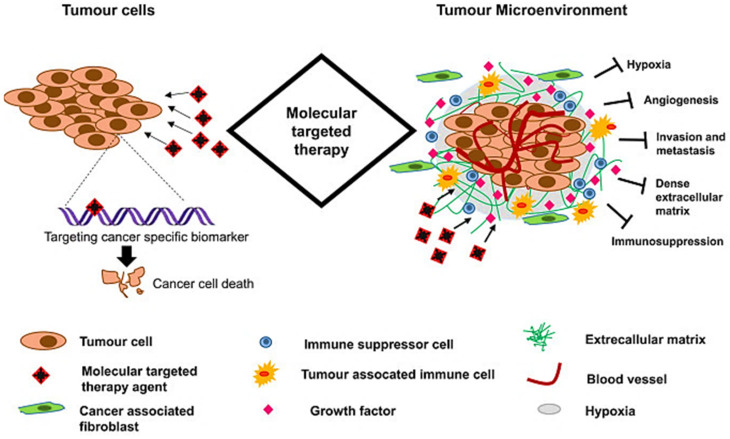
Schematic representation of a molecular targeted therapeutic mechanism for cancer treatment. This focuses on targeting specific cancer-associated molecules that are highly expressed in cancer cells or by modulating the tumor microenvironment (such as tumor vasculature, metastasis, or hypoxia). Reproduced with permission from [147].

Molecular targeted cancer therapy emphasizes targeting specific molecules which are overexpressed in cancer cells by modulating the tumor microenvironment (such as vasculature, metastasis, or hypoxia) [147]. Accordingly, surface modifications of HAp nanostructures for cancer theranostics provide a site-specific drug delivery system, overcoming the side effects of systemic chemotherapy [148]. On this point, understanding the targeting molecules in the desired cancer (Figure 7) is of the utmost importance. For instance, folate receptors (e.g., FRα) are overexpressed in many types of cancer cells; therefore, targeting tumors is possible by using drug delivery systems conjugated with folic acid, a ligand with a high affinity for folate receptors [149]. Folate surface functionalization HAp nanorods were previously reported to effectively target the delivery of doxorubicin (DOX) to a breast cancer MCF-7 cell line [30]. 

**Figure 8:**
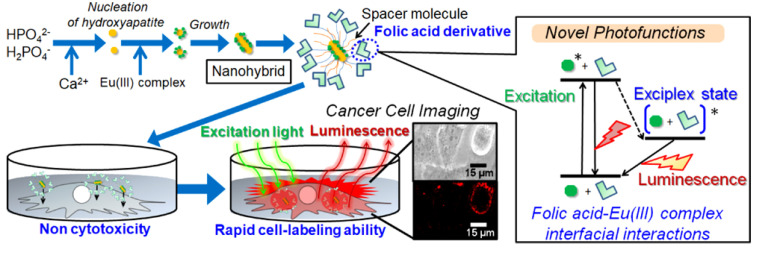
Preparation of luminescent europium (III) complex-based hydroxyapatite nanocrystals (EHA) and evaluation of their cytocompatibility and cell imaging capability. The folic acid derivative, folate N−hydroxysuccinimidyl ester (FA−NHS), was immobilized on the EHA as the targeting ligand for the HeLa cancer cells. Both 3-aminopropyltriethoxysilane and methyltriethoxysilane molecules were utilized for mediation of the immobilization process. Reproduced with permission from [150].

The literature has also confirmed the suitability of surface-engineered HAp nanosystems for imaging cancer cells. For this, Kataoka et al. designed and synthesized efficient luminescent Eu^3+^ complex-based HAp nanocrystals for rapid HeLa cancer cell imaging [150]. HAp nanocrystals were prepared in the presence of tris(2,2,6,6-tetramethyl-3,5 heptanedionato) europium (III) (EuTH) to form inorganic/organic hybrid nanocrystals (named as EHA). After that, the folic acid derivative (folate N-hydroxysuccinimidyl ester (FANHS)) was immobilized on the EHA to target HeLa cancer cells. An effectively dispersible (in phosphate-buffered saline) formulation was obtained after immobilizing positively charged FA-NHS on the samples. Moreover, the intense luminescence from the f−f transition of the Eu^3+^ ions and the charge transfer between the EuTH−FA-NHS exhibited higher quantum efficiency (Figure 8).

## 8. Nanohydroxyapatite-Containing Scaffolds

Generally speaking, biomedical scaffolds act as porous templates allowing for fluid flow (along with nutrients and waste byproducts) and tissue regeneration in 3D once implanted in the body. The “best” characteristics that a scaffold should exhibit (e.g., pore size and distribution and stiffness) indeed depend on the specific tissue to be replaced/regenerated; good matching in terms of the mechanical properties is advisable, as is the need for open and interconnected macropores [151]. Indeed, if the scaffold is intended for cancer treatment, the incorporation of additional, specific functionalities is advisable as well. 

The fabrication of HAp products, such as porous or non-porous blocks, coatings, etc., typically requires a high-temperature stage (sintering) that is key to consolidating ceramic particles to bond together and reduce inter-particle voids. After this heating step, however, the attractive features of nano-sized HAp (e.g., topography, geometry, high specific surface area, etc.) would be irreversibly lost. Therefore, if using nano-sized HAp is a goal, scaffolds should be processed, avoiding the final sintering stage; for this purpose, the incorporation of ceramic NPs within a matrix is a good option, thus yielding composite biomaterials.

The addition of HAp NPs to a biocompatible polymer is the easiest approach to imparting anticancer extra-functionalities to an otherwise biologically inert polymer. In this regard, the first study was reported by Pathi et al. [152], who incorporated HAp NPs, previously obtained by a hydrothermal method, into CO_2_-foamed poly(lactide-co-glycolide) (PLGA) scaffolds that were then seeded with metastatic breast cancer cells. The results showed that smaller, poorly crystalline HAp particles promoted the greater adsorption of adhesive serum proteins and enhanced breast tumor cell adhesion and growth as compared to larger, more crystalline NPs, which, on the other hand, stimulated a higher expression of the osteolytic factor interleukin-8 (IL-8). It was, therefore, suggested that altering the nanoscale properties of the microenvironmental bone mineral plays a critical role in the formation of bone, supporting enhanced cell colonization and growth.

More recently, a group of researchers from Poland carried out a series of in vitro studies on 3D printed poly (L-lactic acid) (PLLA) scaffolds, incorporating HAp NPs doped with europium (III) ions (Eu^3+^). The Eu-doped HAp NPs were prepared by the microwave-stimulated hydrothermal method. A study [153] showed that these composite scaffolds induced apoptosis of an osteosarcoma cell line while not altering the viability of non-transformed adipose-derived human mesenchymal stromal cells. A second study from the same group [154] revealed that PLLA/HAp NPs composite scaffolds enhanced the viability as well as the osteogenic and chondrogenic differentiation potential of adipose-derived human mesenchymal stromal cells. This was associated with an elevated expression of osteogenic and chondrogenic markers on mRNA as well as at the protein level. Moreover, the increased expression of bone morphogenic protein 2 (BMP-2) and BMP-7, along with its receptor (i.e., BMP receptor type 1B (BMPR-1B)), further confirmed the positive effect of these biomaterials on the osteogenic and chondrogenic processes, thus suggesting a possible use in those applications where osseous regeneration is required after bone cancer tumor removal.

It is also worth pointing out that HAp NP-containing polymeric scaffolds have been employed in tumor model studies. Tornin et al. [155] showed the effects of plasma-based therapies on MG-63 cells in a 3D tissue-engineered osteosarcoma model based on a highly porous HAp NPs/collagen scaffold that allowed cancer cell growth and the acquisition of a similar gene expression profile as compared to native tumors. Cancer cells were less sensitive to treatment with cold plasma-activated Ringer’s solution when grown in the 3D scaffold-based model as compared to 2D cultures because the 3-dimensionality of the scaffold induced the expression of a number of protective genes against reactive oxygen and nitrogen species in MG-63 cells, thus favoring cell proliferation and adaptation to oxidative stress. 

HAp NP-containing scaffolds were also used to model non-osseous cancers, such as neuroblastoma. For this purpose, Gallagher et al. [156] mimicked the neuroblastoma microenvironment by using neuroblastoma cell lines and collagen-based scaffolds that were supplemented with either HAp NPs or glycosaminoglycans, which naturally occur in bone and bone marrow, the most common metastatic sites of neuroblastoma. These composite scaffolds allowed neuroblastoma cell attachment, proliferation, and migration as well as the formation of cell clusters, thus eliciting a cell response that was more reflective of the in vivo environment than the 2D cultures. Furthermore, this scaffold-based culture system maintained higher cell densities than the conventional 2D cell culture.

Zhou et al. [103] reported the anticancer properties of composite bio-papers (2D scaffolds) obtained via the self-assembling of Se-doped HAp nanowires (produced by a one-pot solvothermal method) and chitosan. The bio-papers exhibited high flexibility and manufacturability, being easily folded or cut by scissors into various shapes and sizes, inhibiting the growth of chondrosarcoma (HCS-2/8) and osteosarcoma (SJSA) cells more significantly than normal human bone marrow stromal cells (hBMSCs). The anticancer effect was suggested to be associated with the accumulation of ROS and the activation of tumor cell apoptosis. Specifically, the Se-doped HAp/chitosan composite bio-papers killed HCS-2/8 and SJSA cells by simultaneously inducing JNK activation and STAT3 inhibition, thereby promoting cell apoptosis. Furthermore, in vivo studies confirmed the ability of this composite bio-paper to suppress the growth of xenograft tumor models (osteosarcoma) in mice.

Nakayama et al. [157] reported that the self-organization of HAp with poly(acrylic acid) (PAA) leads to the formation of liquid-crystalline hybrid nanorods with appealing properties as a drug release platform for the photodynamic therapy of cancer (methylene blue was used as a model drug). Furthermore, the 2D-oriented scaffolds obtained via spin-coating were shown to induce cellular alignment and elongation along the direction of the hybrid nanorods. 

Jiang et al. [158] developed a complex scaffold by alternatively assembling polydopamine (PDA)-hybridized nano-sized zeolitic imidazolate framework-8 (pZIF-8 nano MOFs) and PDA-decorated-nHAp particles on the surfaces of 3D-printed gelatin scaffolds through a PDA-assisted layer-by-layer assembly strategy. The synthesis of the pZIF-8 nano MOFs relied on mussel-inspired catechol chemistry, which provided the nano MOFs with versatile adhesiveness, high drug loading efficiency, good physiological stability, and tumor environment-sensitive degradability. The pZIF-8 nano MOFs were physiologically stable and acted as intelligent nanocarriers for the double encapsulation and release of large-size osteoinductive BMP-2 and small-size anticancer cisplatin.

HAp NPs-mediated photonic nanomedicine was also explored in other studies; for example, the photothermal therapy of cancer can be enabled when HAp NPs are combined with non-polymeric materials such as graphene oxide (GO). Ma et al. [159] developed a multifunctional scaffold with the double aim of killing cancer cells through thermal ablation and promoting in situ osteogenesis. Specifically, the effect of HAp NPs/GO composite particles with different proportions was investigated on human osteosarcoma cells (HOS), pre-osteoblastic MC3T3-E1 cells, and human bone marrow mesenchymal stem cells (hBMSC), with or without 808 nm near-infrared (NIR) light irradiation. Then, a lyophilized chitosan scaffold was coated with HAp NPs/GO (nHAp: GO = 3:7 by weight) particles, and the resulting composite biomaterial was shown to both effectively kill HOS under 808 nm NIR irradiation by reaching a temperature of 48 °C and to further promote osteogenesis of hBMSC at 42 °C. This scaffold had the best post-operative bone volume/tissue volume ratio performance (20.36%) after 8 weeks of implantation in the cranial defects of rats. Furthermore, NIR irradiation promoted a hemostatic effect as well as the osteogenesis of hBMSC with the addition of HAp NPs by enhancing the BMP2/Smad signaling pathway. 

The same double functionality (photothermal anticancer effect + osteogenesis) of the nano-sized HAp (20 wt.%)/GO composite scaffolds obtained by hydrothermal reaction followed by freeze-drying was reported by Li et al. [160]. In vitro, nano-sized HAp/GO scaffolds killed all but 8% of osteosarcoma cells (MG-63) under 808 nm NIR irradiation for 20 min. Tumor xenografts implanted with the scaffolds in mice reached 60 °C after 4 min of irradiation and stopped growing or even decreased in size after photothermal therapy. Furthermore, micro-tomographic and histological assessments confirmed that nano-sized HAp/GO scaffolds promoted bone regeneration in rat cranial defects: at 8 weeks, new tissue grew by over 65% in the cranial defect area for the scaffold-implanted group, while only 20% regeneration was observed for the control (empty defect). Furthermore, the bone mineral density of the scaffold-implanted group reached about 285 mg/cm^3^, indicating new bone mineral deposition, while only 96 mg/cm^3^ was found for the control.

Apart from polymer/nano-sized HAp and graphene/nano-sized HAp scaffolds, the promise of combining metal and nanostructured HAp was also reported by Zhang et al., who coated titanium cylindrical scaffolds produced by selective laser sintering (porosity 65%, pore size 500 μm) with a layer of wet-synthesized HAp nano-rods (length 46.6 nm, width 13.3 nm). Before undergoing the coating procedure, the titanium scaffolds were acid-alkali treated to form a microporous TiO_2_ network on the surface, thus enhancing bonding with nano-HAp. Then, the porous metal was immersed in a slurry containing HAp nano-rods, methylcellulose, and H_2_O_2_ and left to dry; such a coating procedure was replicated three times, and the scaffolds were eventually heat treated at 300 °C for 1 h. The authors reported in vitro and in vivo studies proving the multifunctional ability of nHAp particles, which acted as both a bone-regenerating material and an antitumor agent. The inhibition of tumor growth, the prevention of metastases, and the enhancement of the survival rate of tumor-bearing rabbits treated with HAp nano-rods were clearly demonstrated. Specifically, the nanomaterial activated mitochondrial-dependent tumor apoptosis in vivo and stimulated an anticancer immune response. Interestingly, histological observations over 1 month suggested that metastasis to the lung could be effectively prevented by using HAp nano-rods but not by using micro-sized HAp or nano-sized TiO_2_ coatings as controls; abnormalities were not found in the liver, heart, kidney, and spleen, thus further supporting the biosafety of the nano-rods being used. These results are in perfect agreement with and expand the findings of a study by Pathi et al. [152] concerning the role of HAp NPs in influencing cancer cell growth cycles.

## 9. Nano-Sized HAp in Cancer Imaging 

Presently, several biomedical imaging techniques are being applied for the detection of all phases of cancer growth. In fact, multiple aspects of cancer, including morphological, structural, metabolic, and functional information, can be identified by applying imaging techniques. These techniques are divided into those applying: (I) non-ionizing electromagnetic radiation (near-infrared spectroscopy, electrical impedance spectroscopy, tomography (EIT), magnetic resonance imaging (MRI), etc.) and (II) ionizing radiation (gamma rays, X-rays or ultraviolet light, computerized tomography (CT), and positron emission tomography (PET)) [161]. The selection of suitable contrast agents is crucial to visualize tumors using specific imaging techniques, such as MRI. During the last several decades, nanotechnology-based materials as contrast agents have been developed and applied for cancer imaging. In this manner, distinct types of nano-sized HAp have been utilized for the molecular imaging of solid tumors. Luminescent, magnetic, and luminomagnetic HAp NPs show appropriate characteristics for the optical, magnetic resonance (MR), and multimodal imaging of cancers [61,162,163]. 

**Figure 9:**
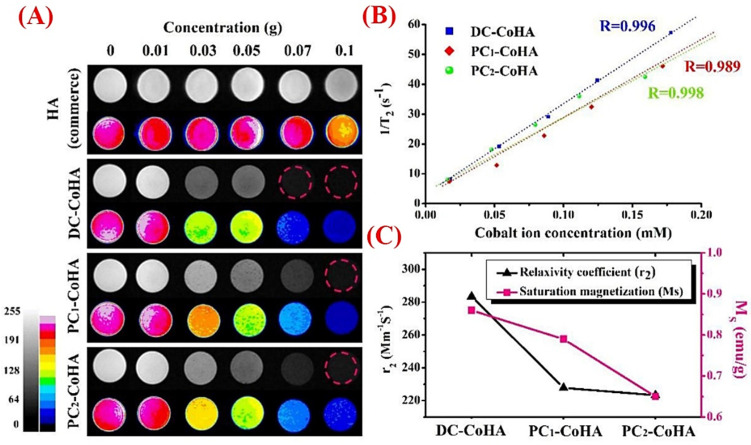
(**A**) T2 weight magnetic resonance imaging (MRI) of commercial HAp (commerce) and the synthesized cobalt (Co)-doped HAp (CoHA) in three groups: synthesized CoHA via direct current (DC), pulse current (PC1) (same time as DC), and PC2 (same voltage as DC). Graph exhibiting the enhanced contrast of the MRI image along with an increase in cobalt concentrations. (**B**) Graph showing the results of the relaxation rate, R2 (1/T2), and T2-weighted spin-echo sequence in the MRI of the HAp doped with different Co concentrations. (**C**) Graph displaying the relaxation rate of CoHA particles, in which the relaxation rates of DC−CoHA, PC1−CoHA, and PC2−CoHA are about 283.4, 227.8, and 223.3 L/mmol s, respectively. Adapted from [166].

The contrast agents used for MRI are commonly divided into (I) positive (T1, Gd-based agents, and brightness contrast) and (II) negative (T2, Fe-based agents, and darkness contrast) types. Hydrophilic Gd(III)-based chelates have been identified as the most widely used agents for MRI to improve diagnosis accuracy. However, concerns still remain about the long-term safety of these materials for the human body [164]. Over the years, inorganic NPs have been revealed as appropriate contrast agents for MRI due to their unique features, such as large surface area and efficient contrasting effect [165]. Among them, superparamagnetic iron oxide NPs (SPIONs) have attracted much attention as MRI contrast agents. However, SPIONs as contrast agents suffer from the accumulation of Fe in soft tissues. The addition of contrast agents to HAp NPs was proposed as a wise method for overcoming all of the aforementioned limitations. For example, Fe-doped HAp NPs were previously reported as suitable contrast agents capable of generating a higher contrast for MRI as compared with SPIONs [43]. In addition to MRI, ^99m^Tc-MDP-labeled Fe-doped HAp NPs were shown to be a proper scintigraphy imaging agent for PET and single-photon emission computed tomography (SPECT) [43]. The substitution of other elements (e.g., cobalt) was also shown to be useful in preparing paramagnetic HAp NPs for use as MRI contrast agents for cancer imaging (Figure 9) [166]. 

**Figure 10:**
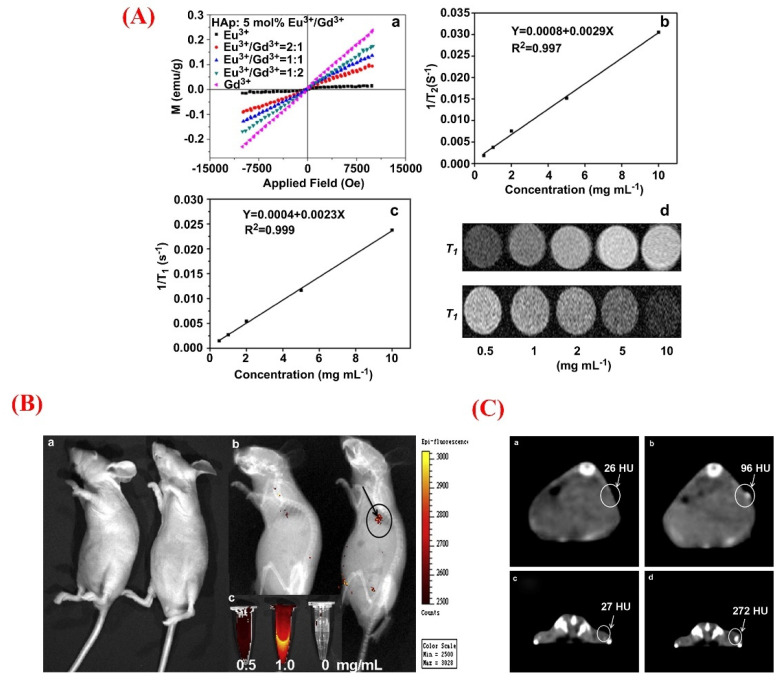
(**A**) Magnetization curves of Eu^3+^/Gd^3+^−HAp nanorods (a); *T*_1_- and *T*_2_- relativity plot of Eu^3+^/Gd^3+^−HAp nanorods (b and c, respectively); *T*_1_- and *T*_2_-weighted magnetic resonance (MR) micrographs of Eu^3+^/Gd^3+^−HAp nanorods dispersed in solution at different concentrations (0.5 to 10 mg mL^−1^) (d). (**B**) In vivo photoluminescence (PL) imaging of the mice at (a) before and (b) after the subcutaneous of injection Eu^3+^/Gd^3+^−HAp (Eu^3+^:Gd^3+^ = 1:2) nanorods; (c) the PL emission micrographs of Eu^3+^/Gd^3+^−HAp nanorods at different concentrations, excited at a wavelength of 430 nm. (**C**) In vivo computed tomography (CT) images of the nude mice treated with Eu^3+^/Gd^3+^−HAp (Eu^3+^:Gd^3+^ = 1:2) nanorods suspended in phosphate-buffered saline solution (PBS); the transverse image of the back of the mouse at (a) before and (b) after the injection; the transverse micrographs of the buttock of the mouse at (c) before and (d) after the injection. Adapted with permission from [167].

The luminomagnetic HAp may provide a highly promising multimodal imaging probe for MRI imaging. In this sense, multifunctional Eu^3+^/Gd^3+^ dual-doped HAp nanorods were previously synthesized via the microwave-assisted method [167]. After being subcutaneously injected into nude mice, this system (Eu^3+^ to Gd^3+^ ratio 1:2, Ms = 0.15) showed proper characteristics as a T1 and T2 contrast agent of MRI as the attenuation value showed an enhancement from 26 to 96 HU (Figure 10). The photoluminescence intensity was dependent on the Eu^3+^/Gd^3+^ ratio in the HAp nanorods, and the magnetization of the samples showed an increase, along with higher concentrations of the Gd^3+^ dopant. It should be highlighted that bio-nanoplatforms made of doped HAp NPs and other materials, such as carbon dots, were previously reported as appropriate tools for cancer cell imaging, which can provide bright blue fluorescence under UV illumination with excellent photostability and colloidal stability [168].

## 10. Concluding Remarks and Future Directions 

HAp has dramatically expanded its range of biomedical applications beyond bone repair over the last few years, showing great promise in the field of cancer theranostics. HAp with nanoscale dimensions is well suited to interact with biomolecules in cells and tissues, as well as drugs, due to its polar surface. This property, along with the biocompatibility and biodegradability of nano-sized HAp upon contact with body fluids, carries the potential for targeted cell- and tissue-specific applications, including cancer therapy and imaging (diagnosis). Multifunctional HAp nanostructures, which potentially combine hyperthermia, drug delivery, photothermal/photodynamic therapy, imaging, and, in general, advanced targeting capabilities, are more versatile and safer than both metallic NPs, which have a solid core and often unknown kinetics of biodegradability, and metal oxide NPs such as SPIONs, which are currently the unique commercial nano-sized product with FDA approval for cancer hyperthermia treatment. We cannot ignore that nanotheranostics is still an emerging research field that has not yet met, in most cases, the minimum required standards for clinical translation. This fact arises from the complexity and synergistic mechanisms of the material(s) used for such multipurpose applications. Indeed, the higher the complexity of the system (e.g., the use of nanostructured HAp as a vehicle for the dual release of ions and drugs with anticancer properties), the higher the number of parameters to be taken into account and, hence, the higher the difficulty in uncoupling the therapeutic effects or quantifying the actual extent of synergistic contributions.

The combination of nano-sized HAp with natural anticancer pharmaceutics, as well as the potential of metallic ion doping and co-doping, are research areas deserving further exploration in the attempt to reduce the use of conventional chemotherapeutics, which usually elicit toxic effects in the body. Furthermore, the incorporation of drugs or ions carrying additional extra-functionalities besides the anticancer effect (e.g., antibacterial or anti-inflammatory properties) is a smart strategy to treat cancer comorbidities, such as infections, which could take place at the bone curettage site. The release of ions and drugs could also be finely modulated by applying functionalization treatments or stimuli-responsive polymeric coatings (acting as molecular gates) onto the surface of HAp NPs.

Going from the nano- to the micro-/macro-scale, the application of additive manufacturing technologies to process HAp NPs will be very helpful in fabricating high-precision products and 3D porous scaffolds with great control over shape, size, and pore characteristics. Three-dimensional printing will also allow the obtaining of composite scaffolds where HAp NPs are embedded in a polymeric gel, thereby expanding the range of functionalities (e.g., incorporation of drugs within the polymeric matrix, finer modulation of mechanical properties, etc.). The customization and personalization of implants will also be possible if 3D printing is combined with medical imaging to reproduce the anatomy of patient’s defects left after cancer removal. 

A more in-depth understanding of the biomolecular impact of nano-sized HAp-based systems on different types of cancer is needed more than ever to allow the research to progress; the experimental work addressed to this purpose is quite complex, involving not only the collaboration of biomaterials scientists, biomolecular chemists, biologists, and oncologists, but also requiring the assessment of full in vitro/in vivo toxicity profiles along with the relationships to the stated hallmarks of cancer. 

## Data Availability

Data are included within this article.
